# Perfluoroalkyl Substances (PFAS) Affect Inflammation in Lung Cells and Tissues

**DOI:** 10.3390/ijms24108539

**Published:** 2023-05-10

**Authors:** Julie Dragon, Michael Hoaglund, Appala Raju Badireddy, Greylin Nielsen, Jennifer Schlezinger, Arti Shukla

**Affiliations:** 1 Department of Pathology and Laboratory Medicine, Larner College of Medicine, University of Vermont, Burlington, VT 05405, USA; julie.dragon@med.uvm.edu (J.D.); mike.s.hoaglund@gsk.com (M.H.); raju.badireddy@uvm.edu (A.R.B.); nielseng@bu.edu (G.N.); jschlezi@bu.edu (J.S.); 2 Department of Environmental Health, School of Public Health, Boston University, Boston, MA 02118, USA

**Keywords:** PFAS, lung, inflammasome, inflammation

## Abstract

Adverse lung outcomes from exposure to per-and polyfluoroalkyl substances (PFAS) are known; however, the mechanism of action is poorly understood. To explore this, human bronchial epithelial cells were grown and exposed to varied concentrations of short-chain (perfluorobutanoic acid, perflurobutane sulfonic acid and GenX) or long-chain (PFOA and perfluorooctane sulfonic acid (PFOS)) PFAS, alone or in a mixture to identify cytotoxic concentrations. Non-cytotoxic concentrations of PFAS from this experiment were selected to assess NLRP3 inflammasome activation and priming. We found that PFOA and PFOS alone or in a mixture primed and activated the inflammasome compared with vehicle control. Atomic force microscopy showed that PFOA but not PFOS significantly altered the membrane properties of cells. RNA sequencing was performed on the lungs of mice that had consumed PFOA in drinking water for 14 weeks. Wild type (WT), PPARα knock-out (KO) and humanized PPARα (KI) were exposed to PFOA. We found that multiple inflammation- and immune-related genes were affected. Taken together, our study demonstrated that PFAS exposure could alter lung biology in a significant manner and may contribute to asthma/airway hyper-responsiveness.

## 1. Introduction

Per- and polyfluroalkyl substances (PFAS) are a large suite of industrial chemicals used in many commercial products (e.g., fabric, cookware and food container coatings), and in aqueous film-forming foams used in firefighting [1,2,3]. Chemically, these are fluorinated carbon chains that have different functional groups, high chemical and thermal stability [4] and surfactant-like properties [5,6,7]. Their extensive use and persistence have led to PFAS becoming ubiquitous environmental contaminants. PFAS from industrial sources and consumer product use are increasingly detected in air, water, soil, and indoor environments [1,8,9,10,11,12] and are capable of long-distance transport [13,14,15]. PFAS, particularly the long-chained forms, bio-accumulate with the highest concentrations detected in the liver and blood. PFAS are also known to reach distal organs (e.g., bone and lung) after oral exposure [16,17,18,19]. PFAS are implicated in developmental, metabolic, autoimmune, reproductive and kidney disorders as well as cancer, type 1 diabetes and celiac disease [20,21,22,23,24,25]. While long-chain (≥C8) PFAS have been phased out of production in the U.S., alternative PFAS (e.g., GenX) have taken their place. Significant and ubiquitous body burdens of both legacy and alternative PFAS in Americans are evident [26], which demands more data on the adverse health effects of diverse PFAS.

Systemic, as well as inhaled, PFAS target the lung and are reported to modify lung surfactant function and pro-inflammatory responses [17,27,28,29,30,31,32]. An association between PFAS, asthma, airway hyper-responsiveness (AHR) and inflammation has previously been reported [33,34,35,36]. Many studies have identified a positive association between exposure to PFAS and asthma-related outcomes in children [8,37] with some inconsistencies [9,38]. A recent study of 675 adolescents in Norway suggests that total PFAS serum concentrations are associated with the occurrence of asthma, while total perfluorooctane sulfonic acid (PFOS), linear PFOS, and linear perfluorohexane sulfonate (PFHxS) double the odds of asthma [39]. A study of 743 children showed a correlation between recurrent respiratory tract infection (RTI) and perfluorobutane sulfonic acid (PFBS) [40]. PFOS and perfluorooctanoic acid (PFOA) accumulate in lung epithelial cells by associating with phospholipids [41]. The literature clearly illustrates the impact of PFAS on lung diseases; however, the mechanistic studies are still limited and need further exploration.

Considering that PFAS act as surfactants and immune modulators [42], we proposed the hypothesis (model) that PFAS exposure would alter lung-cell membrane permeability resulting in NOD-like receptor (NLR) and apoptosis-associated speck-like protein 3 (ASC/PYCARD) (NLRP3) inflammasome activation and pro-inflammatory cytokine upregulation leading to inflammation, asthma or AHR. Using human bronchial lung epithelial cells (BEAS2B), mouse models and PFAS of diverse structures, we demonstrate that PFAS alone or in a mixture can have an immunomodulatory effects on lungs, which may be responsible for the observed lung pathogenesis.

## 2. Results

### 2.1. PFOA Significantly Altered Membrane Properties in BEAS2B Cells

Atomic force microscopy (AFM) performed on PFOS/PFOA exposed BEAS2B cells showed that PFOA exposure significantly altered the plasticity, force ratio and elastic modulus of cells compared to DMSO control (Table 1). PFOS did not have a significant effect on membrane properties.

### 2.2. PFOA Activates NLRP3 Inflammasome in BEAS2B Cells

**Figure 1:**
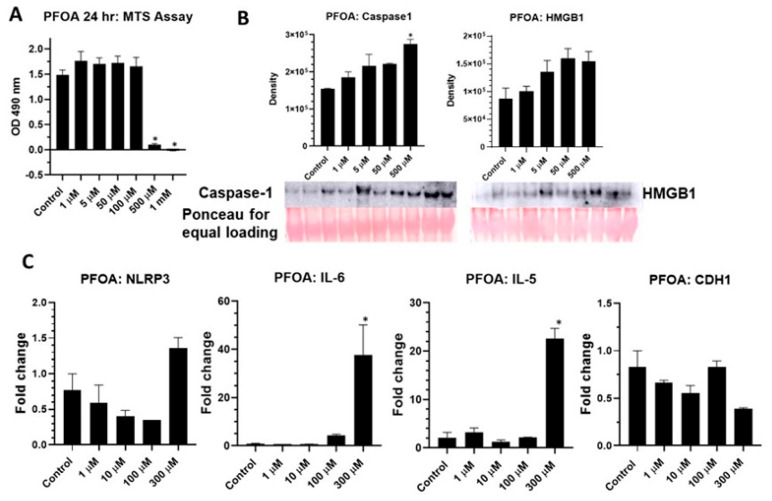
PFOA activates inflammasomes in BEAS2B cells. (**A**) PFOA-attenuated cell viability at 24 h at higher doses as measured by MTS Assay (*n* = 6, * *p* ≤ 0.05 compared to control). (**B**) PFOA-caused inflammasome activation as measured by caspase-1 and HMGB1 secretion in media by immunoblotting. Ponceau stain was used as an equal loading control (*n* = 2/group, * *p* ≤ 0.05 compared to control). Bar graph represents quantitation of immunoblots. (**C**) PFOA-caused increasedsteady-state mRNA levels of NLRP3 inflammasome, pro-inflammatory cytokine IL-6, allergy related cytokine IL-5 and decreased levels of CDH1, a marker of EMT as measured by qRTPCR normalized to hprt (*n* = 2, * *p* ≤ 0.05 compared to control). Equal volume of DMSO was used in control in all experiments.

BEAS2B cells were exposed to a range of concentrations of PFOA (1–1000 μM) for 24 h, and cell survival was measured by MTS Assay compared to DMSO control. Higher concentrations of PFOA caused significant cell death (Figure 1A). A range of concentrations of PFOA were then tested to see the effects on NLRP3 activation and priming. Figure 1B shows the release of caspase-1 and HMGB1 in the media at all concentrations of PFOA tested; however, significance was only reached at the highest concentration, suggesting that the activation of NLRP3 was a response to PFOA exposure. Increases in steady-state levels of *NLRP3*, *IL-6* and *IL-5* mRNAs (Figure 1C) demonstrated the effect of PFOA on NLRP3 priming and pro-inflammatory/allergic cytokine upregulation. A decreasing trend observed in steady-state mRNA levels of E-cadherin (*CDH1*) by PFOA could indicate a possible role for PFOA in epithelial-to-mesenchymal transition (EMT) (Figure 1C).

### 2.3. PFOS Activates NLRP3 Inflammasome in BEAS2B Cells

**Figure 2:**
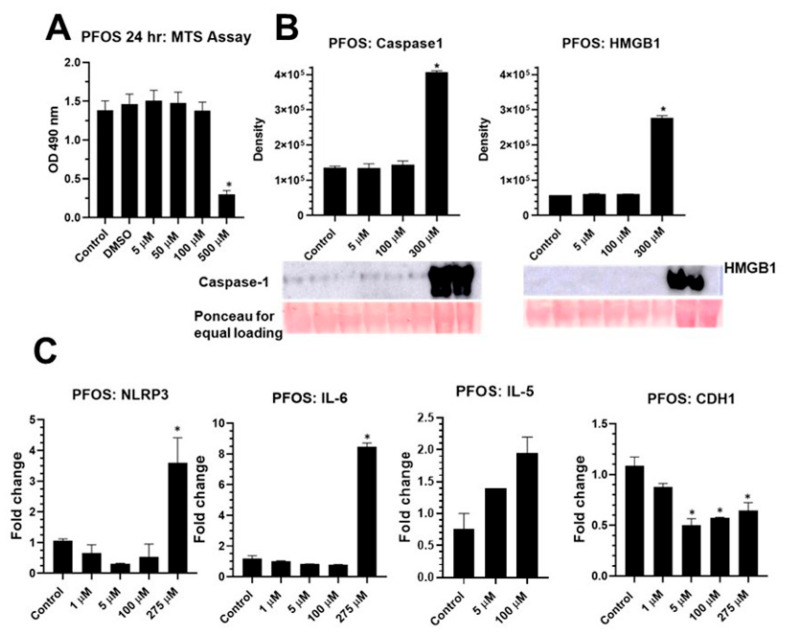
PFOS activates inflammasome in BEAS2B cells. (**A**) PFOS-attenuated cell viability at 24 h at higher doses as measured by MTS Assay (*n* = 6, * *p* ≤ 0.05 compared to control). (**B**) PFOS-caused inflammasome activation as measured by caspase-1 and HMGB1 secretion in media assessed by immunoblotting. Ponceau stain was used as an equal loading control (*n* = 2/group, * *p* ≤ 0.05 compared to control). Bar graphs represent quantitation of immunoblots (**C**). PFOS caused an increase in steady-state mRNA levels of NLRP3 inflammasome, pro-inflammatory cytokines IL-5 and 6 and decreased levels of CDH1, a marker for EMT as measured by qRTPCR (*n* = 2, * *p* ≤ 0.05 compared to control). Equal volume of DMSO was used in control in all experiments.

BEAS2B cells were exposed to a range of concentrations of PFOS (1–500 μM) for 24 h, and cell survival was measured by MTS Assay compared to DMSO control. The effect of DMSO (≤0.01%) alone on cell viability was also measured and found to have had no significant effect (Figure 2A). Higher concentrations of PFOS caused significant cell death (Figure 2A). A range of concentrations of PFOS were then tested to determine the effects on NLRP3 activation and priming. Figure 2B shows the release of caspase-1 and HMGB1 in the media at a high concentration of PFOS; quantitation by density assessment showed a significant effect (Figure 2B). Increases in steady-state mRNA levels of *NLRP3*, *IL-6* and *IL-5* (Figure 2C) demonstrated the effect of PFOS on NLRP3 priming and pro-inflammatory/allergic cytokine levels. A significant decrease observed in steady-state mRNA levels of *CDH1* by PFOS indicated a possible role for PFOS in initiating the EMT process (Figure 2C).

### 2.4. Effect of Short Chain and a Mixture of Long and Short Chain PFAS on BEAS2B Cell Viability

**Figure 3:**
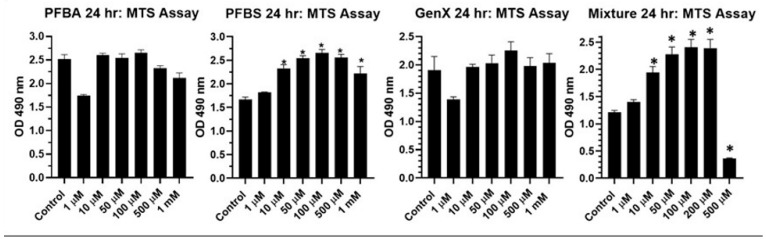
Effect of individual short-chain (PFBA, PFBS, GenX) or long- and short-chain mixture (PFOA, PFOS, PFBA, PFBS and GenX) on BEAS2B cell viability as measured by MTS Assay. * *p* ≤ 0.05 compared to control (*n* = 6/group).

As long-chain PFAS (PFOA, PFOS) are being replaced by short-chain PFAS (PFBA, PFBS, GenX) and the fact that the human population is exposed to a mixture of PFAS [25], we conducted experiments with short-chain PFAS as well as with a mixture of PFAS. As presented in Figure 3, the short-chain PFBA and GenX had no significant effect on cell viability. However, PFBS induced a significant increase in viability in a dose-dependent manner. The mixture of all five PFAS at low concentrations also increased viability, but the highest concentration reduced viability (Figure 3). This was an interesting observation. Because an MTS assay measures mitochondrial activity, it is possible that PFBS did not increase the proliferation/growth of the cell but simply the metabolism via mitochondrial activity.

### 2.5. PFAS Mixture Activates NLRP3 Inflammasome and Pro-Inflammatory Cytokines in Lung Epithelial Cells

**Figure 4:**
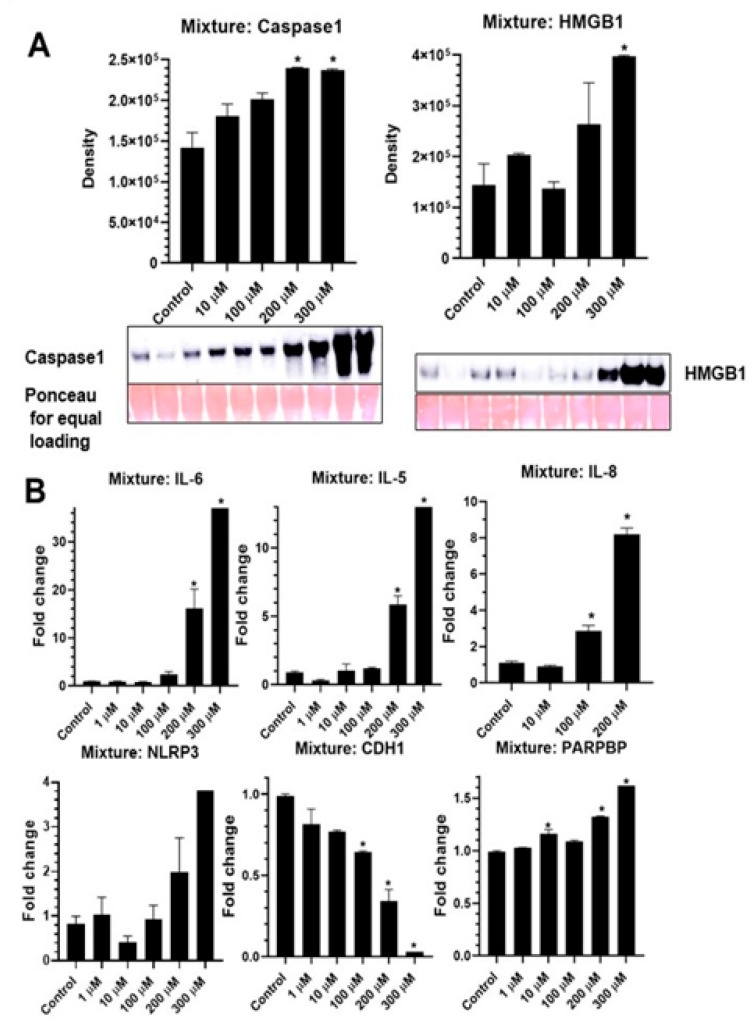
PFAS mixture causes inflammasome activation in BEAS2B cells as depicted by caspase-1 and HMGB1 release in media (**A**). An increase in steady-state mRNA levels of *NLRP3*, *IL-6*, *IL-5* and *PARPBP* and decrease in *CDH1* levels were caused by mixture (PFOA, PFOS, PFBA, PFBS and GenX) exposure (**B**). * *p* ≤ 0.05 compared to control. (*n* = 2/group).

A mixture of 5 PFAS (short and long chain) caused significant increases in caspase-1 and HMGB1 release in media, a measure of NLRP3 activation (Figure 4A). The mixture also resulted in increased steady-state mRNA levels of *NLRP3*, *IL-6*, *IL-5*, and *IL-8*, demonstrating its effect on NLRP3 priming and pro-inflammatory signals in BEAS2B cells (Figure 4B). The steady-state mRNA levels of proliferation related gene, PCNA-interacting partner (*PARPBP*) also increased (Figure 4B), which may have played a role in the observed increased viability with some short-chain PFAS as presented earlier. The EMT marker *CDH1* was again downregulated by PFAS mixture as was seen before with individual PFAS (Figure 4B), confirming that EMT plays a significant role in PFAS-induced lung pathogenesis.

### 2.6. In Vivo Studies

The concentrations of PFOA in treated drinking water were 1.4 and 6.2 mg/L. Based on average daily consumption (0.21 mL/g mouse/day), the daily exposures were approximately 0.3 and 1.2 mg/kg/day. This resulted in serum concentrations of 29 ± 8 and 107 ± 15 μg/mL, respectively (*n* = 3–6). Male and female mice did not show significant differences in serum levels of PFOA after 14 weeks of consumption.

### 2.7. RNA Seq-Gene Expression on Mouse Lung Tissues Exposed to PFOA

WT mice lungs exposed to a high concentration of PFOA in water showed alterations in 62 protein coding genes (Table 2, FDR < 0.2, *p* > 0.05, and 2× fold change) 59 of which are depicted.

**Figure 5:**
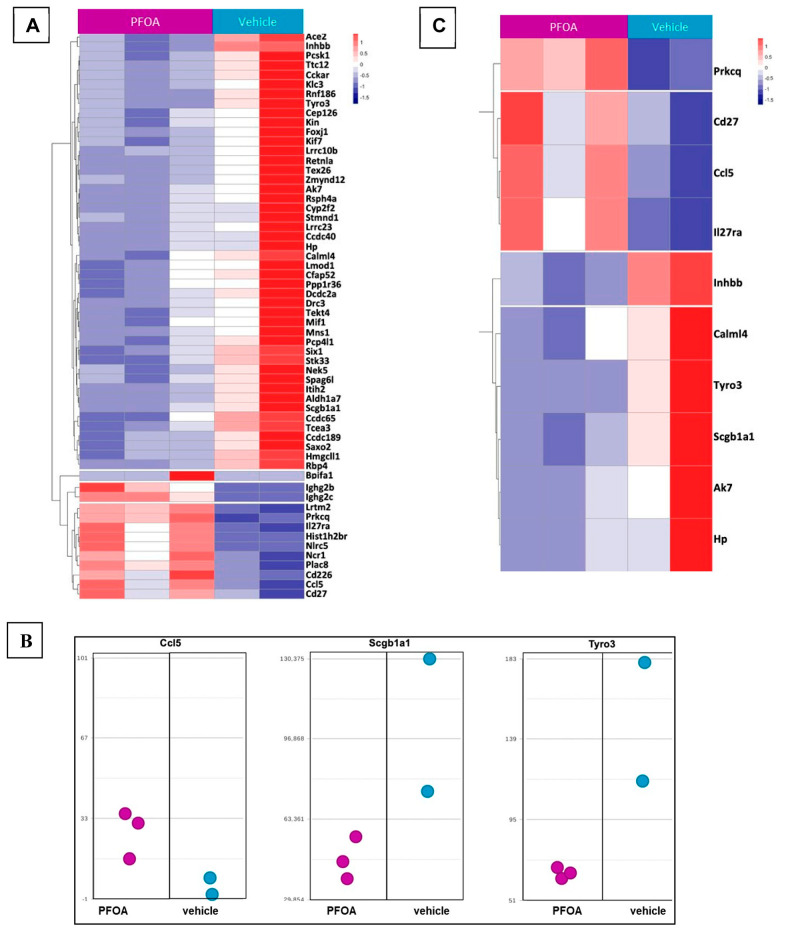
(**A**) Heat map showing effect of administering PFOA-treated drinking water (6.2 µg/mL) on mice for 14 weeks in differential gene expression in lungs compared to vehicle group (V) as measured by Next Gen Sequencing (NGS). (**B**) Dot plots of the key players from GO: 0050727 “regulation of inflammatory response” from the DEG set. (**C**) Heatmap of the raw counts for the GO: 0050727 “regulation of inflammatory response” and other inflammatory gene sets.

In Figure 5A. it should be noted that only one WT vehicle sample could not be included in these analyses due to RNA quality issues and weak sequencing results. Gene-set enrichment analysis identified a number of cytokines and immunity-related genes enriched due to exposure to PFOA. Gene-set enrichment analysis of the differentially expressing genes (DEG) for this comparison showed that *Ccl5*, *Tyro3*, *Hp*, *Ak7*, *Cd27*, *Prkcq* and *Scgb1a1* were the key players in GO: 0050727 “regulation of inflammatory response” (Figure 5B, *p* > 0.016, Table 3). Genes involved in this enrichment cluster are represented in Figure 5C as a heatmap.

**Figure 6:**
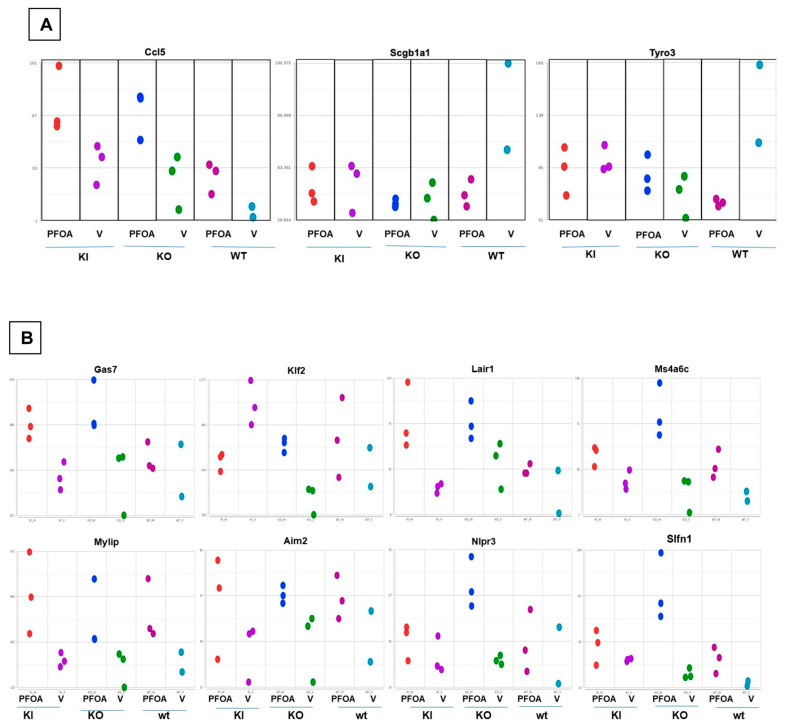
Gene analysis across all genotypes (WT, KI, KO) with and without PFOA. (**A**). Dot plots of the key players from GO: 0050727 “regulation of inflammatory response” from the DEG set, including the PPARα knock-out and knock-in (KO & KI respectively). (**B**). Dot plots of differentially expressed genes in KO and KI in response to PFOA or Vehicle, (V). (*n* = 3), (WT = wild type; KI = expression of hPPARα; KO = deletion of PPARα).

The knock-out of mouse PPARα (KO) altered 71 protein-coding genes while human PPARα (KI) affected a single protein-coding gene in response to PFOA consumption (FDR < 0.2, *p* > 0.05, and 2× fold change, see Supplemental Table S1). The combined analyses of the abovementioned 3 genes (*Ccl5*, *Tyro3*, *Scgb1a1*) in 6 groups repeated the findings in WT mice, whereas KO or KI had no significant effect on these genes (Figure 6A). The other top differentially expressed genes in combined analyses were *Col1α1*, *Gas7*, *Klf2*, *Lair1*, *Lrg1*, *Mfsd2a*, *Mylip*, *Scd1*, *Slfn4*, *Slfn1*, *Ms4a6c*, *Ripk3*, *Nlrp3*, *Aim2*. The comparison of gene expression in the three genotypes (WT, KO, KI) with and without PFOA can be seen in Figure 6B. The differences in expression patterns across genotypes suggested different roles for PPARα in controlling gene expression in the lung and warrants detailed investigation.

## 3. Discussion

PFAS exposure by air, water or food is a significant public health problem [25] associated with asthma and AHR in a number of human cases and in animal studies. It is important to understand the mechanism(s) of PFAS-induced lung pathogeneses such as asthma/AHR so that biomarkers or therapeutic targets can be identified. Our research here is a step in this direction.

Inflammasomes are considered to play an important role in asthma and allergic diseases as evidenced from population, mouse-model and cell-based studies [43,44,45,46]. Inflammasomes, multi-protein platforms comprised of nucleotide-binding oligomerization domains, control the activation of the cysteinyl aspartate protease, caspase-1 and the cleavage of pro-IL-1β, which enables the release of the active mature IL-1β cytokine [47] along with IL-18 and HMGB1 [48]. *NLRP3* is the most studied inflammasome and is known to be activated by various particles/fibers including asbestos [47,48]. A growing number of studies have demonstrated the association of NLRP3 and PFAS in gastric cells [29] and rodent models of obesity and lung development [30,31]. Our experience with lung pollutants, the inflammasome field and the published literature led us to hypothesize that PFAS, being a surfactant in nature, could alter cell-membrane permeability, leading to potassium efflux and inflammasome activation. Consistent with the findings of Sorli et al. [27], we demonstrated the alteration of the membrane properties of BEAS2B cells by PFOA. Subsequent activation of NLRP3, as measured by caspase-1 and HMGB1 release in the medium, occurred following exposure to PFOA, PFOS and 5 PFAS mixture (long and short chain). Increased steady-state mRNA levels of pro-inflammatory and -allergic cytokines signified a role for PFAS in lung inflammation and pathogenesis. A recent comprehensive study by Wang et al. [28], using in vitro macrophages and in vivo mouse models indicated the role of another inflammasome, AIM2, in PFOS-induced inflammatory responses but no involvement of NLRP3 in the process. The discrepancy in the outcome could be attributed to the use of different cell types (epithelial vs. macrophage) to test the effects of PFOS or differences in the effects of structurally distinct PFAS.

The viability assessment of BEAS2B cells in response to different doses of PFAS projected an interesting picture. Both long-chain PFAS, PFOA and PFOS had no effect on viability up to a concentration of 100 μM; however, higher concentrations produced significant cell death. NLRP3 activation and priming also were significantly increased at higher concentrations, which suggested that cells were undergoing pyroptosis (inflammasome-dependent inflammatory cell death). Of the short-chain PFAS, PFBA had no effect on cell viability at any concentration; however, PFBS significantly increased viability and GenX showed an increasing trend. Similarly, PFBS has recently been reported to increase cell viability in trophoblasts as well [49]. Furthermore, it is possible that both PFBS and GenX did not increase the viability or growth of cells but only their metabolic activity because the MTS Assay used here for cell viability measures mitochondrial activity.

As the human population is exposed to a mixture of PFAS, it is important to assess the role of a mixture of short- and long-chain PFAS on biological cell pathways [25]. Our 5 PFAS mixture had a significant effect on NLRP3 activation and priming in BEAS2B cells. Pro-inflammatory (IL-6, IL-8) and pro-allergic (IL-5) cytokines were also upregulated. E-cadherin (CDH1), a marker for EMT was downregulated both by PFOA and PFOS alone as well as in a PFAS mixture. EMT is considered an important initial step for many respiratory diseases including asthma and fibrosis [50,51].

PFOA consumption in drinking water is a relevant model for PFOA exposure that mimics real-life situations. Our model mimicked high human-relevant PFAS exposure (range 0.01–92.03 μg/mL serum). Here we showed that chronic exposure to PFOA increased serum levels of PFOA and altered the lung gene-expression profile in mice expressing wildtype PPARα. Many of these genes are involved in immunity/inflammation pathways, suggesting that systemic PFOA exposure can alter lung gene expression, which may lead to lung pathogenesis. We focused on 3 genes (out of 10), *Ccl5*, *Tyro3*, and *Scgb1a1*, which were significantly altered by PFOA exposure in the WT group. These genes are the key players in GO: 0050727 “regulation of inflammatory response”. C–C motif chemokine ligand 5 (CCL5) is a pro-inflammatory chemokine known to be involved in respiratory infection and diseases including lung cancer [52,53,54]. The secretoglobulins (SCGB) are highly abundant in the respiratory system and regulate immunoregulatory and anti-inflammatory process of airway diseases. Their downregulation by PFOA (Figure 5B) can stimulate disease process in lungs [55]. Tyro 3 is a component of the TAM receptor (family of receptor tyrosine kinases) along with Axl and MerTK. TAM plays important roles in efferocytosis and balancing the immune response and inflammation [56]. In different immune cells, TAM can prevent superfluous immune reactions and dampen the inflammatory response. Decreased levels of Tyro 3 in mouse lung by PFOA indicated a possible mechanism by which PFOA could regulate the lung’s immune/inflammatory system. A recent study by Phelps et al. [57] using zebrafish and human neutrophils demonstrated that legacy and that emerging PFAS can suppress the neutrophil respiratory burst, thereby suppressing the immune function.

Interestingly, we saw increased expression of two inflammasome-related genes, *Nlrp3* and *Aim2*, in response to PFOA exposure in the mouse lung. It corroborated our in vitro data that one possible mechanism of lung inflammation and pathogenesis by PFAS was inflammasomes. Similarly, Wang et al. showed that in vivo exposure to IP-injected PFOS resulted in Aim2-dependent inflammation. Detailed studies are required with lung specific transgenic models to understand the role of different inflammasomes in PFAS-induced lung pathologies. The following were all upregulated or had an upregulation trend in PFOA-consuming mice lungs compared to vehicle-fed mice: *Klf2* (kruppel-like factor 2), a transcription factor involved in type I pneumocyte differentiation; *Mfsd2a* (major facilitator superfamily domain containing 2a), known to maintain pulmonary surfactant homeostasis; *Mylip* (myosin-regulated light-chain interacting protein) involved in protein catabolic process and possibly as a tumor suppressor in lung cancer; and *Slfn4 & 1* (schlafen 4 and 1), *Ms4a6c*, involved in immune cell regulation, cell cycle arrest and receptor signaling pathways,

Although it is a very preliminary finding from a limited sample size, PFOA consumed through water was found to affect the lung immune/inflammatory environment, which may play a significant role in lung pathogenesis, asthma and AHR. Downward trends in the PFOA-exposed mice group were also observed in genes such as, *Col1α1* (collagen type1α1), *Gas 7* (growth arrest specific 7) and *Scd* 1 (stearoyl-coenzyme A desaturase 1); however, their significance is yet to be determined.

One of the strongest responses to PFAS exposure is lipid disruption [25,58], which can be mediated by peroxisome proliferator activated receptor α (PPARα) and acts in a species-specific manner. Therefore, we compared PFOA-modulated gene expression profiles in lung tissues from PPARα null (KO) and hPPARα (KI) mice to that in WT mice. No significant effect of PPARα manipulation (KI, KO) was observed for *Ccl5*, *Lair1*, *Mylip* and *Aim2*, all of which appeared to be induced in the lung by PFOA. However, the PFOA-induced reduction of *Tyro3* and *Scgb1a1* expression appeared only to occur in the WT mice. In contrast, *Gas7*, *Ms4a6c*, *Nlrp3* and *Slfn1* showed greater induction by PFOA in the absence of PPARα. *Klf2*, which showed an upward trend in response to PFOA in WT and KO mice, was reduced by PFOA in KI mice. These different patterns suggested that PFOA may have multiple molecular targets in the lung as has been shown for the liver [59].

**Figure 7:**
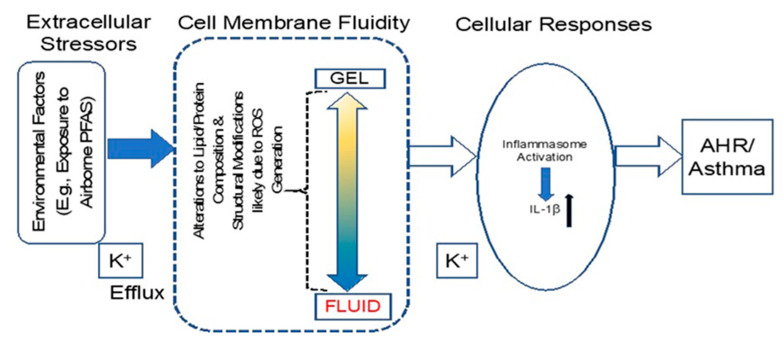
PFAS can alter lung epithelial cell membrane fluidity/permeability, K+ efflux, reactive oxygen species (ROS) generation, which may lead to inflammasome activation, pro inflammatory cytokine (IL-1β, IL-18, IL-33) release and airway hyper-responsiveness (AHR)/asthma.

In conclusion, our study demonstrated that PFAS can dysregulate lung inflammasome/inflammation/immune pathways via membrane permeability alterations which may be responsible for the PFAS-associated respiratory diseases reported (Figure 7). Both short- and long-chain PFAS alone as well as in mixture were shown to affect lung biological responses. In addition, the number of inflammation- and immunity-related genes in the lungs of mice were altered in response to PFOA-treated drinking water. We acknowledge that there were limitations to our study, such as the use of one type of lung cell line, lack of inclusion of a primary cell line and a limited number of samples for the in vivo study. The high points of our study were testing the effect of both the short- and long-chain PFAS and the mixture of PFAS in the in vitro study and using a human-relevant mouse model that allowed us to compare the effects between mice expressing mouse or human PPARα and PPARα-null mice. The role of PPARα in PFOA-induced lung gene expression may not be as significant as in the liver or adipose tissue where lipid metabolism takes place; however, the results here support the need for further investigation of the mechanistic underpinning of PFAS-induced effects on lung health. More studies are required to pinpoint the role of inflammasome/inflammation in PFAS-induced lung responses using transgenic, lung-specific animal models.

## 4. Materials and Methods

Cell culture and treatments: Immortalized BEAS2B cells from ATCC [60,61] were cultured following protocols previously published [11,60,61]. PFAS proposed in this study were purchased from commercial vendors (Sigma-Aldrich, Burlington, MA, USA). Cells were exposed to long-chain (PFOA, PFOS), short-chain PFBS, perfluorobutanoic acid (PFBA), replacement chemical for PFOA (GenX), or mixture (all 5) PFAS for 24 h at predetermined concentrations.

Atomic force microscopy (AFM) was performed on BEAS2B grown on special glass dishes, exposed to PFOA/PFOS or vehicle (DMSO) for 24 h. Alteration in membrane properties (plasticity, elastic modulus and force ratio) were measured (*n* = 89–100 measurements/dish). Cells grown on glass bottom dishes were analyzed using AM–FM AFM (Asylum Research MFP-3 D BIO (MIC, UVM)] to quantify the fluidity of cellular membranes) [62,63].

PFAS effects on cell viability by MTS Assay: Dose-response analyses were conducted to assess the viability of the cell line following exposure to a range of PFAS individually as well as in mixture. Stock solutions of PFAS in DMSO were diluted in 0.5% fetal bovine serum (FBS) containing a culture medium for cell exposure (24 h). Cells were exposed to a broader range of concentrations to cover the range of the human body burden (0.01–92.03 µg/mL) [64]. The same volume of DMSO was used as a vehicle control (≤0.01%).

The effects of PFAS were seen in NLRP3 priming (mRNA levels), NLRP3 activation (caspase-1 and HMGB1 secretion in medium), cytokine mRNA expression (*IL-5* and *IL-6*) and EMT marker mRNA expression (E-cadherin, *CDH1*). Lung epithelial cells were exposed to PFOA, PFOS or mixture of 5 PFAS (1:1:1:1:1) for 24 h with a range of selected concentrations.

Priming of inflammasome *NLRP3*, *AIM2*, *PYCARD*, *IL-1β*, *gasderminD*: This was assessed on extracted RNA by qRTPCR with assay-on-demand (AOD) primer and probe mixture from Applied Biosystems [48,51,60] normalized to housekeeping gene *HPRT*.

Activation of inflammasome: A conditioned medium was collected from dishes after 24 h of treatment and high-mobility group box 1 (HMGB1), and caspase-1p20 secretion by Western blot analysis was assessed as previously reported [48,51]. Ponceau stain was used to demonstrate equal loading.

Statistical analyses: We used three or more determinations per group per time point, and experiments were replicated three times. GraphPad Prism 8 (GraphPad Software, La Jolla, CA, USA) was used. Analysis of variance (ANOVA) was used to evaluate the results from each experiment, followed by a Tukey’s post-hoc test to assess the combined results from replicate experiments. The latter analysis included an experiment as a random effect to test for differences between experiments and treatment by experimental interactions. The Student–Newman–Keuls procedure was used to adjust for multiple pairwise comparisons between groups (*p* ≤ 0.05 was considered statistically significant).

In vivo exposure: PFOA-exposed and control mouse lungs (frozen) were obtained from Dr. Jennifer Schlezinger (BU), who had previously demonstrated the effect of PFOA-treated drinking water in mice using different models [59,65]. Perfluorooctanoic acid, PFOA (cat. #171468, 95% pure) came from Sigma-Aldrich (St. Louis, MO, USA). All animal studies were approved by the Institutional Animal Care and Use Committee at Boston University and performed in an American Association for the Accreditation of Laboratory Animal Care accredited facility (Animal Welfare Assurance Number: A3316-01). Male and female, humanized PPARα (KI) and PPARα null (KO) mice were generated from mouse PPARα null, and human PPARα heterozygous breeding pairs (generously provided by Dr. Frank Gonzalez, NCI) [66]. Sv/129 wildtype (WT) mice were purchased from Jackson labs, Bar Harbor, ME (stock #002448). At 6 weeks of age, mice were provided a custom diet based on the “What we eat in America (NHANES 2015/2016)” analysis for what adults eat (Research Diets, New Brunswick, NJ, USA) [67]. The diet contained 47% carbohydrate, 37% fat, and 16% protein, as a % energy intake. Vehicle and treatment water were prepared from NERL High Purity Water. A concentrated stock solution of PFOA (1 × 10^−2^ M) was prepared in NERL water and then diluted in NERL water containing 0.5% sucrose. Mice were administered vehicle (0.5% sucrose) drinking water or PFOA (1.4 (low) and 6.2 (high) μg/mL) drinking water *ad libitum* for 14 weeks. Vehicle water was prepared with 0.5% sucrose, which ensures consumption. Treatment water was prepared with vehicle water and PFOA stocks dissolved in water. Treatment was for 14 weeks. At the time of harvest, lungs were removed and snap frozen in liquid nitrogen and transported to UVM in dry ice. Serum PFOA concentrations were determined by LC-MS/MS according to method MLA-110 (EPA Method 537 Modified) (SGS AXYS Analytical Services Ltd., Sidney, British Columbia, CA, USA).

Next Gen Sequencing (RNA Seq) on mouse lungs: Lung tissue RNA extraction, quantification, quality analyses, library preparation, sequencing and data analyses was performed by the Vermont Integrative Genomics Resource (VIGR), UVM. Libraries were sequenced across four lanes of a high-capacity flow cell on the Illumina HiSeq 1500. Demultiplex reads had poorer quality bases trimmed from the 3′ end (phred score < 20), and the trimmed reads (avg. quality > 38.1, avg. length 80 bp, avg. GC ~53.4%) were aligned to the mouse reference genome mm10 using the STAR 2.6 aligner in Partek^®^ Flow^®^. Aligned reads were then quantified using an expectation–maximization model and translated to genes. Those that had fewer than 20 counts were then filtered, leaving 19,366 high-count genes. Differential gene expression was evaluated using the algorithm in DESeq2 v3.5 base on median ratio normalization, and the negative binomial distribution of the gene signal. Gene-set enrichment analysis and pathway enrichment analysis were performed against the KEGG gene sets and pathways databases.

## Figures and Tables

**Table 1:** PFAS alters BEAS-2B cell membrane properties as measured by atomic force microscopy (AFM). (SEM-standard error of mean).

Treatment	Plasticity			Force Ratio			Elastic Modulus (Pa)		
	Mean	SEM	n	Mean	SEM	n	Mean	SEM	n
DMSO	0.276	0.0217	89	5.299	0.4668	95	521,803	41,398	97
PFOS	0.238	0.0158	96	5.013	0.2219	95	602,628	38,920	100
PFOA	0.305	0.0801	95	2.921	0.2770	95	699,743	159,913	96

**Table 2:** Differentially expressed protein-coding genes from the lungs of wild type (WT) mice exposed to PFOA compared to those exposed to vehicle (V) based on an FDR < 0.05, *p*-value < 0.05, and 2× fold change cut off. Due to the limited number of available vehicle-treated lungs, several genes including *Bpifa1*, *Ccdc40*, *Hp* and *Stmnd1*, artifactually passed these thresholds despite weak support and validation on independent samples.

Ensembl	Chr	Total Counts	*p*-Value	FDR (Step-Up)	Fold Change
Bpifa1	2	946	5.29 × 10^−9^	9.88 × 10^−5^	2.36 × 10^6^
Ighg2c	12	232	1.34 × 10^−4^	9.61 × 10^−2^	9.80 × 10^1^
Hist1h2br	13	85	9.02 × 10^−4^	1.87 × 10^−1^	7.89 × 10^1^
Ighg2b	12	234	6.84 × 10^−4^	1.75 × 10^−1^	1.46 × 10^1^
Ncr1	7	551	8.33 × 10^−4^	1.87 × 10^−1^	9.14 × 10^0^
Ccl5	11	663	2.69 × 10^−4^	1.32 × 10^−1^	5.59 × 10^0^
I830077J02Rik	3	371	9.65 × 10^−5^	7.36 × 10^−2^	5.18 × 10^0^
Plac8	5	745	7.30 × 10^−4^	1.75 × 10^−1^	3.86 × 10^0^
Cd27	6	453	3.25 × 10^−4^	1.38 × 10^−1^	3.32 × 10^0^
Cd226	18	873	2.47 × 10^−4^	1.32 × 10^−1^	3.30 × 10^0^
Il27ra	8	647	6.11 × 10^−4^	1.70 × 10^−1^	2.65 × 10^0^
Lrtm2	6	1051	2.07 × 10^−4^	1.21 × 10^−1^	2.41 × 10^0^
Prkcq	2	1757	2.81 × 10^−7^	2.62 × 10^−3^	2.38 × 10^0^
Nlrc5	8	3571	1.09 × 10^−3^	1.96 × 10^−1^	2.01 × 10^0^
Kin	2	2205	6.73 × 10^−4^	1.75 × 10^−1^	−2.03 × 10^0^
Lmod1	1	6101	1.19 × 10^−3^	2.00 × 10^−1^	−2.04 × 10^0^
5330417C22Rik	3	12,012	8.49 × 10^−6^	2.54 × 10^−2^	−2.04 × 10^0^
Kif7	7	1439	4.81 × 10^−4^	1.66 × 10^−1^	−2.07 × 10^0^
Cyp2f2	7	142,500	5.99 × 10^−4^	1.70 × 10^−1^	−2.07 × 10^0^
Inhbb	1	2105	9.52 × 10^−6^	2.54 × 10^−2^	−2.08 × 10^0^
Rnf186	4	1163	8.24 × 10^−4^	1.87 × 10^−1^	−2.10 × 10^0^
Hp	8	44,707	2.96 × 10^−4^	1.38 × 10^−1^	−2.14 × 10^0^
Lrrc10b	19	1435	5.51 × 10^−4^	1.70 × 10^−1^	−2.15 × 10^0^
Cckar	5	3927	7.17 × 10^−5^	6.38 × 10^−2^	−2.18 × 10^0^
Hmgcll1	9	1103	3.40 × 10^−4^	1.38 × 10^−1^	−2.24 × 10^0^
Mns1	9	4942	6.98 × 10^−4^	1.75 × 10^−1^	−2.24 × 10^0^
Drc3	11	2063	1.19 × 10^−3^	2.00 × 10^−1^	−2.26 × 10^0^
Ttc12	9	2140	4.64 × 10^−5^	5.30 × 10^−2^	−2.27 × 10^0^
Aldh1a7	19	9082	1.30 × 10^−5^	2.70 × 10^−2^	−2.28 × 10^0^
Foxj1	11	4375	1.10 × 10^−3^	1.96 × 10^−1^	−2.30 × 10^0^
Rbp4	19	1647	3.00 × 10^−5^	4.54 × 10^−2^	−2.35 × 10^0^
Ccdc189	7	1386	6.25 × 10^−5^	5.83 × 10^−2^	−2.35 × 10^0^
Pcp4l1	1	3787	1.92 × 10^−4^	1.17 × 10^−1^	−2.39 × 10^0^
Lrrc23	6	2829	5.69 × 10^−4^	1.70 × 10^−1^	−2.40 × 10^0^
Ak7	12	5344	8.81 × 10^−4^	1.87 × 10^−1^	−2.42 × 10^0^
Tcea3	4	1315	9.63 × 10^−4^	1.88 × 10^−1^	−2.43 × 10^0^
Scgb1a1	19	910,638	5.78 × 10^−6^	2.54 × 10^−2^	−2.52 × 10^0^
Ccdc40	11	6721	5.28 × 10^−4^	1.70 × 10^−1^	−2.53 × 10^0^
Tyro3	2	1570	2.54 × 10^−5^	4.32 × 10^−2^	−2.55 × 10^0^
Ccdc65	15	945	1.10 × 10^−3^	1.96 × 10^−1^	−2.56 × 10^0^
Cep126	9	3598	9.01 × 10^−4^	1.87 × 10^−1^	−2.56 × 10^0^
Calml4	9	1519	3.79 × 10^−4^	1.42 × 10^−1^	−2.58 × 10^0^
Klc3	7	956	5.11 × 10^−5^	5.30 × 10^−2^	−2.59 × 10^0^
Stk33	7	1254	3.48 × 10^−4^	1.38 × 10^−1^	−2.59 × 10^0^
Rsph4a	10	4311	3.38 × 10^−4^	1.38 × 10^−1^	−2.65 × 10^0^
Ace2	X	1782	5.94 × 10^−5^	5.83 × 10^−2^	−2.69 × 10^0^
Saxo2	7	1942	5.28 × 10^−4^	1.70 × 10^−1^	−2.71 × 10^0^
Spag6l	16	2335	3.38 × 10^−4^	1.38 × 10^−1^	−2.72 × 10^0^
Nek5	8	1539	4.64 × 10^−5^	5.30 × 10^−2^	−2.77 × 10^0^
Ppp1r36	12	987	6.87 × 10^−4^	1.75 × 10^−1^	−2.81 × 10^0^
Dcdc2a	13	1097	6.02 × 10^−4^	1.70 × 10^−1^	−2.81 × 10^0^
Cfap52	11	1362	5.18 × 10^−4^	1.70 × 10^−1^	−2.98 × 10^0^
Tekt4	17	984	2.81 × 10^−4^	1.35 × 10^−1^	−3.12 × 10^0^
Mlf1	3	1739	1.03 × 10^−3^	1.92 × 10^−1^	−3.13 × 10^0^
Six1	12	935	1.03 × 10^−3^	1.92 × 10^−1^	−3.45 × 10^0^
Stmnd1	13	1442	6.27 × 10^−4^	1.70 × 10^−1^	−3.54 × 10^0^
Itih2	2	466	1.59 × 10^−4^	1.06 × 10^−1^	−3.71 × 10^0^
Retnla	16	2736	7.31 × 10^−6^	2.54 × 10^−2^	−5.01 × 10^0^
Pcsk1	13	218	9.91 × 10^−4^	1.91 × 10^−1^	−5.06 × 10^0^
Zmynd12	4	172	5.76 × 10^−4^	1.70 × 10^−1^	−6.57 × 10^0^
Tex26	5	147	9.66 × 10^−4^	1.88 × 10^−1^	−8.53 × 10^0^
Gm3417	17	384	1.20 × 10^−5^	2.70 × 10^−2^	−2.31 × 10^1^

**Table 3:** Gene ontological enrichment analysis filtered by the keywords “inflammatory” and “inflammation”.

Gene Set	Description	Enrichment Score	*p*-Value	Genes in List	Genes not in List
GO: 0050727	regulation of inflammatory response	4.14199	0.0158912	4	286
GO: 0050728	negative regulation of inflammatory response	1.06038	0.346326	1	126
GO: 0050729	positive regulation of inflammatory response	2.98854	0.0503611	2	106
GO: 0002437	inflammatory response to antigenic stimulus	2.52479	0.0800755	1	24
GO: 0002526	acute inflammatory response	1.81842	0.162283	1	52
GO: 0002673	regulation of acute inflammatory response	4.23522	0.0144766	2	53
GO: 0002675	positive regulation of acute inflammatory response	5.54591	0.00390341	2	26
GO: 0002861	regulation of inflammatory response to antigenic stimulus	2.41631	0.0892499	1	27
GO: 0002863	positive regulation of inflammatory response to antigenic stimulus	3.01936	0.0488324	1	14
GO: 0002864	regulation of acute inflammatory response to antigenic stimulus	2.95645	0.0520031	1	15
GO: 0002866	positive regulation of acute inflammatory response to antigenic stimulus	3.32299	0.0360447	1	10
GO: 0006954	inflammatory response	5.10092	0.00609113	5	344

## Data Availability

We have submitted NGS data to NCBI. The GEO number is GSE231602.

## Supplementary Materials

The following supporting information can be downloaded at: https://www.mdpi.com/article/10.3390/ijms24108539/s1.
